# NSUN2 restrains gastric cancer cell apoptosis and ferroptosis by promoting the m5C modification of EPYC

**DOI:** 10.1186/s41065-025-00626-x

**Published:** 2026-01-19

**Authors:** Lei Wu, Boxuan Chen, Si Cheng, Xiaofeng Fang, Fen Zhou

**Affiliations:** 1 Department of Gastrointestinal surgery, Xianning Central Hospital, The First Affiliated Hospital of Hubei University of Science and Technology, No. 228, Jingui Road, Xian ’an District, Xianning City, Hubei Province 437100 China; 2 Department of Gastroenterology, Xianning Central Hospital, The First Affiliated Hospital of Hubei University of Science and Technology, Xianning City, Hubei Province 437100 China

**Keywords:** Gastric cancer, EPYC, NSUN2, M5C modification

## Abstract

**Background:**

Epiphycan (EPYC) has been confirmed to play an oncogenic role in many cancers. However, its role and mechanism in gastric cancer (GC) progression has not been explored.

**Methods:**

The levels of EPYC and NOP2/Sun domain 2 (NSUN2) were detected by qRT-PCR and western blot. Cell proliferation, apoptosis, migration and invasion were determined by cell counting kit 8 assay, colony formation assay, flow cytometry, wound healing assay and transwell assay. Fe^2+^ and iron levels were examined to assess cell ferroptosis. Actinomycin D assay was used to detect the effect of NSUN2 knockdown on EPYC mRNA stability, and methylated RNA immunoprecipitation (MeRIP) assay was performed to determine the effect of NSUN2 silencing on 5-methylcytosine (m5C) level of EPYC. Xenograft tumors were constructed to explore the regulation of NSUN2 knockdown on GC tumorigenesis in vivo.

**Results:**

EPYC was abnormally higher expressed in GC tissues and cells. Knockdown of EPYC restrained GC cell proliferation, migration and invasion, while enhanced apoptosis and ferroptosis. NSUN2 had elevated expression in GC, which could increase the mRNA stability and expression of EPYC through m5C modification. NSUN2 silencing inhibited GC cell proliferation, metastasis, promoted apoptosis and ferroptosis, while these effects were reversed by EPYC overexpression. In vivo experiments revealed that NSUN2 downregulation reduced GC tumorigenesis by decreasing EPYC level in vivo.

**Conclusion:**

NSUN2-mediated m5C modification of EPYC contributed to GC cell growth and metastasis, which provided a novel regulatory axis for understanding the pathogenesis of GC.

**Supplementary Information:**

The online version contains supplementary material available at 10.1186/s41065-025-00626-x.

## Introduction

Gastric cancer (GC) is a disease with high molecular and phenotypic heterogeneity and is the third most common cause of cancer death [1]. With the increasingly serious aging of the global society, the incidence of GC is gradually increasing [2]. For early GC, perioperative chemotherapy has become the standard of care [3]. Unfortunately, GC is usually diagnosed at a late stage and has a poor prognosis [4]. At present, GC is shifting from traditional pathological typing to precision therapy guided by molecular typing, but it still faces challenges such as low early diagnosis rate [5, 6]. Therefore, it is necessary to seek effective and reliable GC therapeutic targets.

Epiphycan (EPYC) is a proteoglycan belonging to the small leucene-rich repeat proteoglycan (SLRP) family [7, 8]. Previous studies have found that EPYC plays a role in many human diseases, including cancer. It has been reported that EPYC was highly expressed in ovarian cancer tissues and could promote cell metastasis and proliferation [9]. Yang et al. detected the upregulated EPYC level in pancreatic cancer cells, and confirmed that EPYC facilitated cancer malignancy progression via accelerating cell proliferation [10]. Therefore, EPYC may be an oncogene in human cancers. Supporting this notion, our study discovered that EPYC was abnormally upregulated in GC tissues through database analysis. However, the role and underlying molecular mechanisms of EPYC in GC progression remain largely unknown.

5-methylcytosine (m5C) is one of the RNA modifications that exist in eukaryotic RNA of different species [11], and has been confirmed to regulate the occurrence of different cancer types [12, 13]. Recent studies have shown that RNA modifications (such as m5C) not only regulate the malignant behavior of tumor cells, but also may interact with the tumor immune microenvironment by affecting programmed cell death (such as apoptosis and ferroptosis), thereby shaping the immunosuppressive phenotype and promoting tumor progression [14, 15]. There is evidence that m5C abnormal modification is associated with the development of GC [16]. NOP2/Sun domain 2 (NSUN2) is one of the mRNA methyltransferases that catalyze m5C modification [17]. Hu et al. reported that NSUN2 was overexpressed in GC tissues, which promoted GC cell metastasis and proliferation via regulating the m5C methylation of PIK3R1 and PCYT1A [18]. Besides, NSUN2 enhanced GC peritoneal metastasis and colonization through increasing the m5C modification of ORAI2 [19]. Our study found that EPYC contained m5C modification sites using RNAm5cfinder software, and further analysis suggested that NSUN2 promoted EPYC m5C level to mediate its mRNA stability. However, whether NSUN2 regulated GC progression by mediating EPYC m5C modification remains unclear.

Based on the above, our study speculated that NSUN2-mediated m5C modification of EPYC facilitated GC malignant behaviors. These findings provide new evidence for understanding the pathogenesis of GC.

## Materials and methods

### Samples

A total of 30 paired GC tissues and adjacent normal tissues were collected from 30 GC patients at Xianning Central Hospital, The First Affiliated Hospital of Hubei University of Science and Technology. Inclusion criteria: diagnosed as GC through histopathological analysis, not treated with surgical resection, chemotherapy or radiation therapy prior to admission, received surgical resection of the primary tumors after admission. Exclusion criteria: patients with mental disorders, infectious diseases, other cancers or a history of treatment for other cancers. All samples were kept in liquid nitrogen until use. Each participant signed the written informed consent, and this study was approved by the Ethics Committee of Xianning Central Hospital, The First Affiliated Hospital of Hubei University of Science and Technology.

### Cell culture and transfection

Human GC cells (AGS and SNU-1) and normal gastric epithelial cells (GES-1), purchased from Biovector NTCC (Beijing, China), were maintained in RPMI-1640 medium containing 1% penicillin/streptomycin and 10% FBS. After reaching 50% confluence, AGS and SNU-1 cells were transfected with shRNAs against EPYC/NSUN2 (sh-EPYC/sh-NSUN2#1/#2) and an EPYC overexpression vector (oe-EPYC) with Lipofectamine 3000 (Invitrogen, Carlsbad, CA, USA). AGS cells were treated with different concentrations of Erastin for 24 h, or 10 µM Erastin for different times, to screen optical dose and time. To confirm the ferroptosis phenotype, AGS cells transfected sh-NSUN2 for 48 h, followed by treated with iron chelators DFO (50 µM) or ferroptosis inhibitor Ferrostatin-1 (Fer-1, 2 µM) for 24 h.

### qRT-PCR

TRIzol reagent (Invitrogen) was used for RNA extraction and cDNA Synthesis Kit (Invitrogen) was utilized for reverse-transcription. qRT-PCR process was performed by mixing SYBR Green (Takara, Tokyo, Japan), cDNA and specific primers (Table 1). Relative EPYC/NSUN2/GPX4/SLC7A11 mRNA level was calculated using 2^−ΔΔCt^ method with β-actin as internal control.

**Table 1:** Primer sequences used for qRT-PCR

Name		Primers for PCR (5’-3’)
EPYC	Forward	CTGTGACTGCCCCAACTCTA
	Reverse	AGAGCCATCAATCAGCCTGG
NSUN2	Forward	GCTACCCCGAGATCGTCAAG
	Reverse	TCAGGATACCTTTTGTAACCAGT
β-actin	Forward	GGATTCCTATGTGGGCGACGA
	Reverse	GCGTACAGGGATAGCACAGC
GPX4	Forward	CTTTGCCGCCTACTGAAGCC
	Reverse	ACTTCGGTCTTGCCTCACTG
SCL7A11	Forward	ACCCTGAAAACCAGGCTGAC
	Reverse	TCAAGCAGCAGTACAGCACA

### Western blot (WB)

After extracted by RIPA buffer, total proteins were electrophoresed by SDS-PAGE gel and transferred onto PVDF membrane. Then, the membrane was incubated with anti-EPYC (1:1000, H00001833-M06, Abnova, Taipei, China), anti-NSUN2 (1:1000, ab259941, Abcam, Cambridge, CA, USA), anti-GPX4 (1:1000, ab125066), anti-SLC7A11 (1:1000, ab307601), anti-ACSL4 (1:1000, ab155282), anti-β-actin (1:5000, ab179467), and secondary antibody (ab205718 or ab205719). Finally, the membrane was treated with Immobilon Western Chemilum HRP Substrate (Millipore, Billerica, MA, USA), and protein blots were analyzed by ImageJ software.

### Cell counting kit 8 (CCK8) assay

GC cells in 96-well plates were cultured for 48 h and then incubated with CCK8 reagent (Dojindo, Kumamoto, Japan). After that, cell viability was analyzed at 450 nm by a microplate reader.

### Colony formation assay

Cells were dispensed into 6-well plates (1000 cells/well). After 14 days, the colonies were fixed by paraformaldehyde and stained with crystal violet, after which the number of colonies (> 50 cells) was analyzed under a microscope.

### Flow cytometry

Collected GC cells were suspended with binding buffer and stained with Annexin V-FITC and PI solution (Invitrogen). After 15 min, cell apoptosis rate was analyzed using Cytoflex flow cytometer.

### Wound healing assay

Cells were seeded into 6-well plates and cultured until they reached 90% confluence. Then, a wound was created with a 200 µL pipette tip, and cells were cultured with serum-free medium for 24 h. Images were taken at 0 and 24 h under a microscope, and migration distance was counted to calculate migration ratio.

### Transwell assay

Cells suspended with serum-free medium were added into Matrigel-coated transwell upper chamber (BD Biosciences, San Jose, CA, USA). Completed medium was added into the lower chamber. After 24 h, invaded cells were fixed and stained, and then counted under a microscope.

### Detection of cell ferroptosis

According to the instruction of Iron Assay Kit (ab83366, Abcam), the levels of intracellular Fe^2+^ and total iron were analyzed in GC cells. Lipid ROS level was analyzed using C11-BODIPY Lipid Peroxidation Assay Kit (S0043S, Beyotime).

### Actinomycin D assay

Cells transfected with sh-NC/sh-NSUN2 were treated with 5 µg/mL actinomycin D solution (Abcam) for 0, 8, 16 and 24 h. At each time point, cells were collected for detecting EPYC mRNA level by qRT-PCR.

### Methylated RNA Immunoprecipitation (MeRIP) assay

For the high-scoring 1294 m5C sites, sh-NSUN2-WT/MUT and oe-EPYC-WT/MUT were constructed. Total RNAs isolated from cells transfected with sh-NC/sh-NSUN2, sh-NSUN2-WT/MUT or oe-EPYC-WT/MUT were fragmented, and then treated with protein A/G magnetic beads, anti-IgG or anti-m5C for immunoprecipitation using Magna MeRIP mRNA Methylation Kit (Millipore). The m5C modification level of EPYC was analyzed by qRT-PCR.

### Animal models

AGS cells were stably infected with lentivirus sh-NC/sh-NSUN2 and then injected into the right flank of BALB/c nude mice (*n* = 5/group, SPF Biotechnology Co., Ltd., Beijing, China). Tumor volume was detected every 5 days and mice were euthanized after 25 days. The collected tumor tissues were used for immunohistochemical (IHC) staining and qRT-PCR after being photographed and weighed. Animal study was approved by the Animal Ethics Committee of Xianning Central Hospital, The First Affiliated Hospital of Hubei University of Science and Technology.

### Statistical analysis

All experiments were performed in triplicate, with each independent experiment set 3 times to generate an average value. Data are interpreted as mean ± SD by GraphPad Prism 8.0 software. The comparisons were performed using Student’s *t*-test (for 2 groups) or ANOVA (for multiple groups) followed by Bonferroni multiple test correction. *P* < 0.05 was considered statistically significant.

## Results

### EPYC was upregulated in GC tissues and cells

**Fig. 1:**
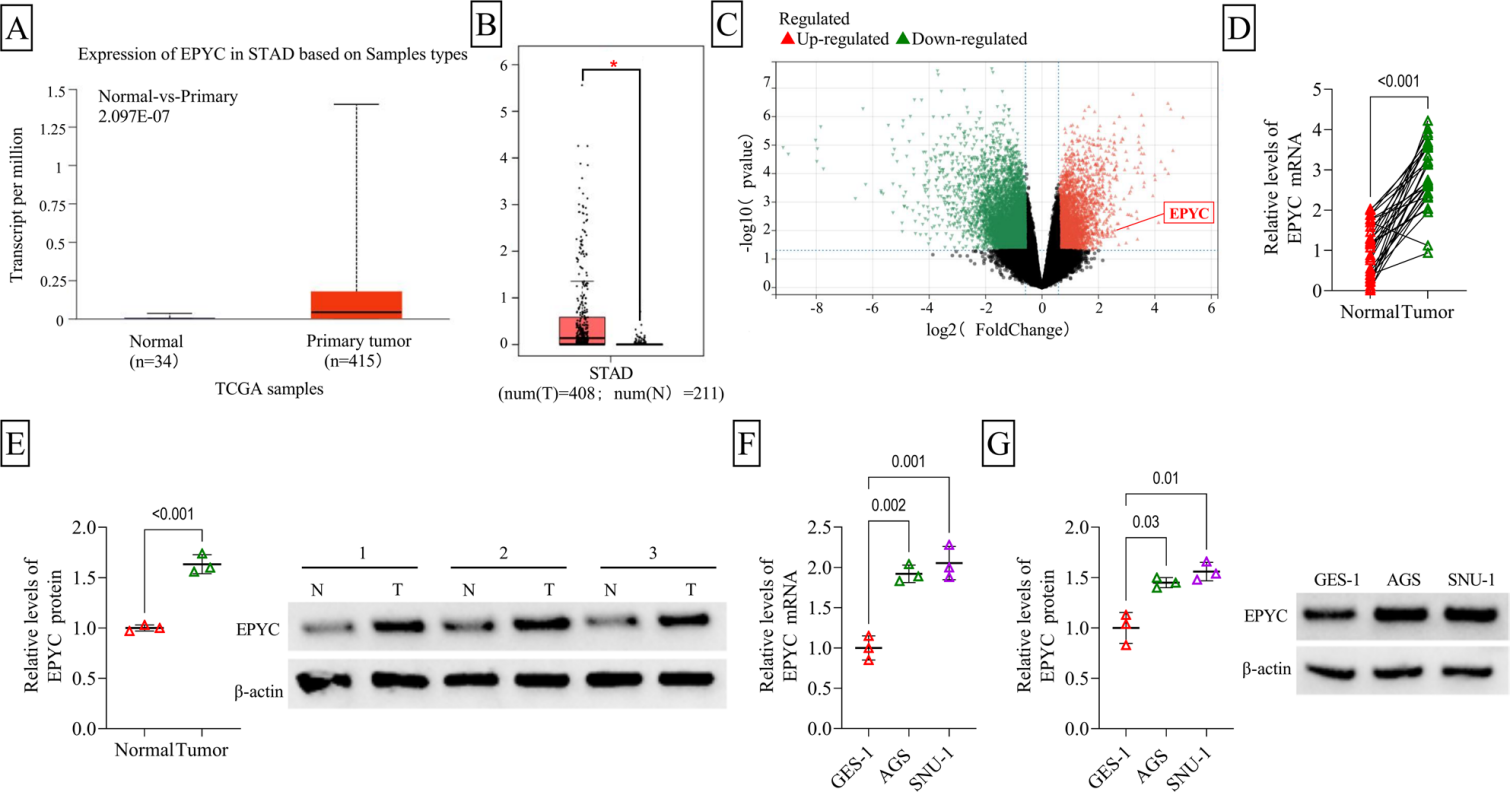
EPYC expression in GC tissues and cells. **A** TCGA database and **B** GEPIA database analyzed EPYC expression in STAD tissues and normal tissues. **C** Volcano plots showed differentially expressed genes in normal tissues and GC tissues in GSE79973 database. **D**-**E** EPYC expression in GC tissues and adjacent normal tissues was detected by qRT-PCR (*n* = 30) and WB (*n* = 3). **F**-**G** qRT-PCR and WB were used to examine EPYC expression in GC cells (AGS and SNU-1) and GES-1 cells (*n* = 3). **D**-**E**, Student’s *t*-test; **F-G**, one-way ANOVA. All experiments were performed in triplicate

According to the prediction of TCGA and GEPIA, EPYC had increased expression in stomach adenocarcinoma (STAD) tissues (Fig. 1A-B). Also, TCGA database analysis showed the expression of EPYC in different TNM stage and the feasibility of EPYC as a diagnostic indicator for STAD (Supplementary Fig. 1A-B). In addition, the GEO database (GSE79973) analyzed the differentially expressed genes in normal and GC tissues, and the volcano plot showed that EPYC was highly expressed in GC tissues (Fig. 1C). Consistent with database analyzing, our study detected the high EPYC mRNA and protein levels in GC tissues compared to adjacent normal tissues (Fig. 1D-E). Besides, EPYC expression also was higher in GC cell lines (AGS and SNU-1) than that in GES-1 cells (Fig. 1F-G).

### Silencing of EPYC accelerated GC cell apoptosis and ferroptosis

**Fig. 2:**
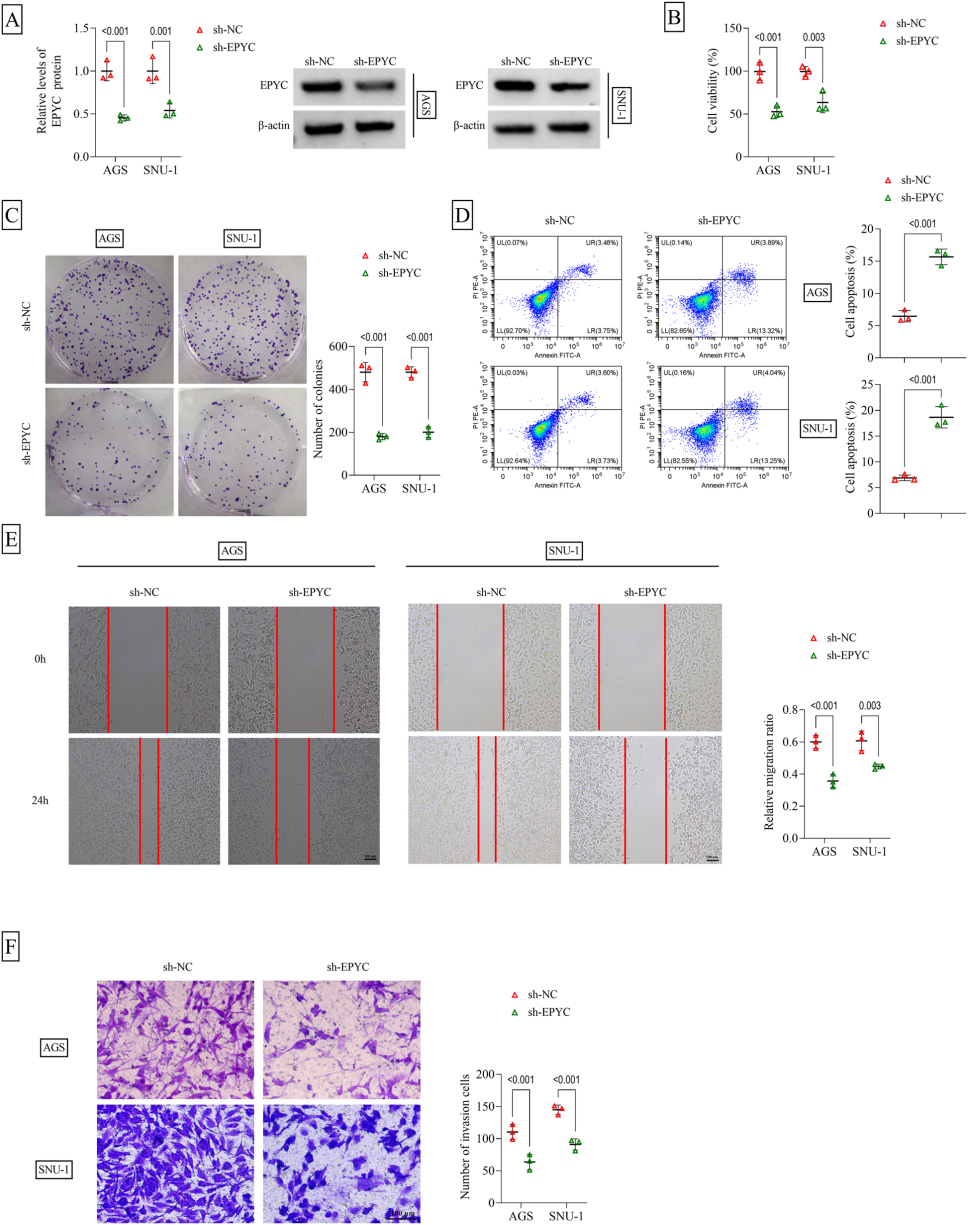
Effect of sh-EPYC on GC cell proliferation, apoptosis and metastasis. AGS and SNU-1 cells were transfected with sh-NC/sh-EPYC (*n* = 3). **A** EPYC protein level was examined by WB. **B** CCK8 assay, **C** colony formation assay, **D** flow cytometry, **E** wound healing assay and **F** transwell assay were used to analyze cell proliferation, apoptosis, migration and invasion. A-C and E-F, two-way ANOVA; D, Student’s *t*-test. All experiments were performed in triplicate

**Fig. 3:**
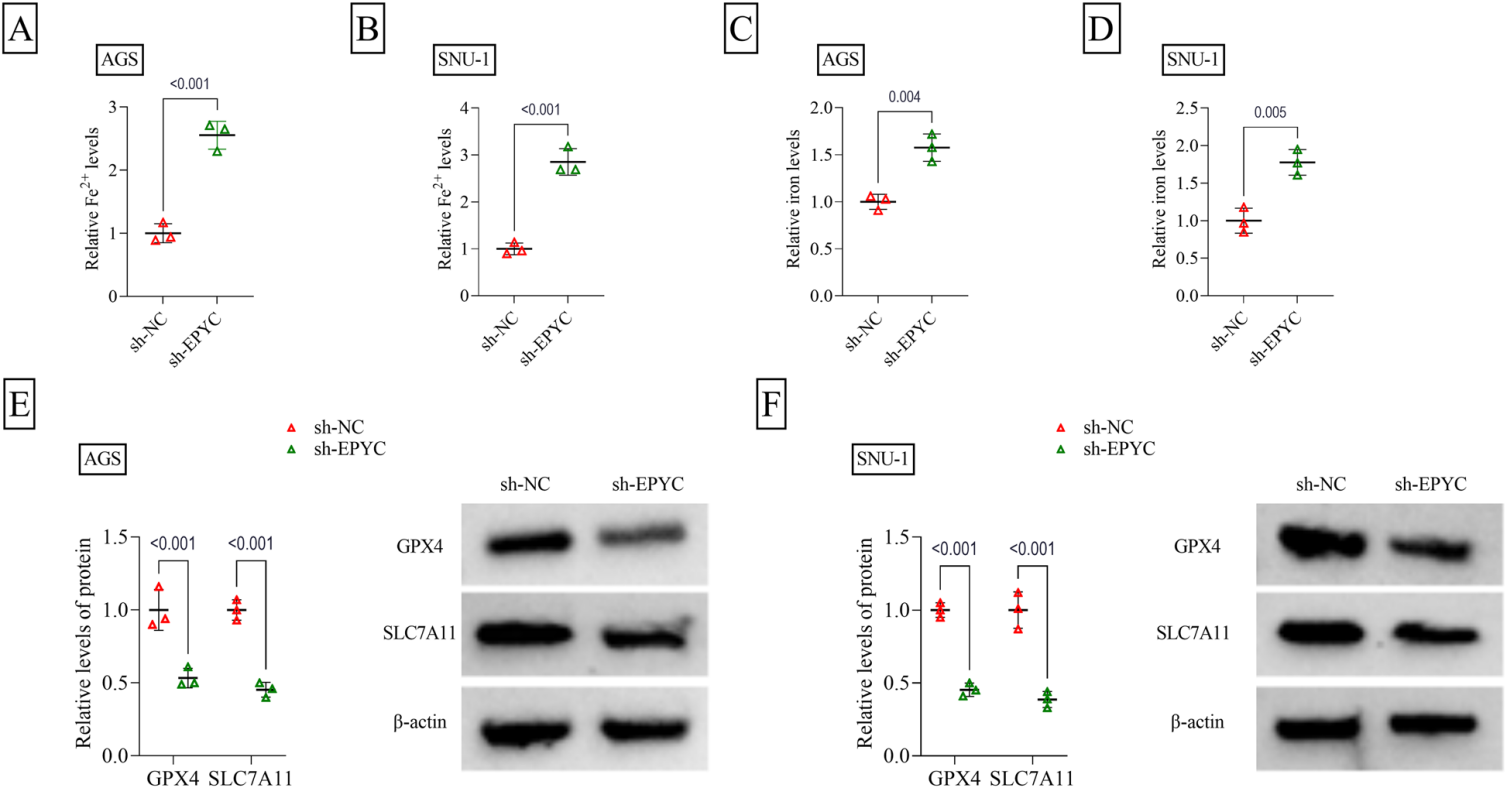
Effect of sh-EPYC on GC cell ferroptosis. AGS and SNU-1 cells were transfected with sh-NC/sh-EPYC (*n* = 3). **A**-**D** Fe^2+^ and iron levels were examined by Iron Assay Kit. **E**-**F** GPX4 and SLC7A11 protein levels were examined by WB. **A**-**D**, Student’s *t*-test; **E**-**F**, two-way ANOVA. All experiments were performed in triplicate

To explore the role of EPYC, knockdown experiments were performed in AGS and SNU-1 cells. After transfected with sh-EPYC, EPYC protein level was markedly reduced in AGS and SNU-1 cells (Fig. 2A). Functional assay showed that EPYC silencing could inhibit cell viability, decrease colony numbers, and promote apoptosis rate in AGS and SNU-1 cells (Fig. 2B-D). Also, knockdown of EPYC suppressed migration ratio and invasion cell numbers in AGS and SNU-1 cells (Fig. 2E-F). The levels of Fe^2+^ and iron could be enhanced by EPYC downregulation (Fig. 3A-D). Also, EPYC knockdown reduced the protein levels of anti-ferroptosis markers (GPX4 and SLC7A11), as well as enhanced lipid ROS level and ferroptosis-marker ACSL4 protein level, in AGS and SNU-1 cells (Fig. 3E-F and Supplementary Fig. 2A-B). EPYC knckdwon Therefore, our study believed that EPYC might promote GC cell proliferation and metastasis, while suppress apoptosis and ferroptosis.

### NSUN2 promoted EPYC mRNA stability by m5C modification

**Fig. 4:**
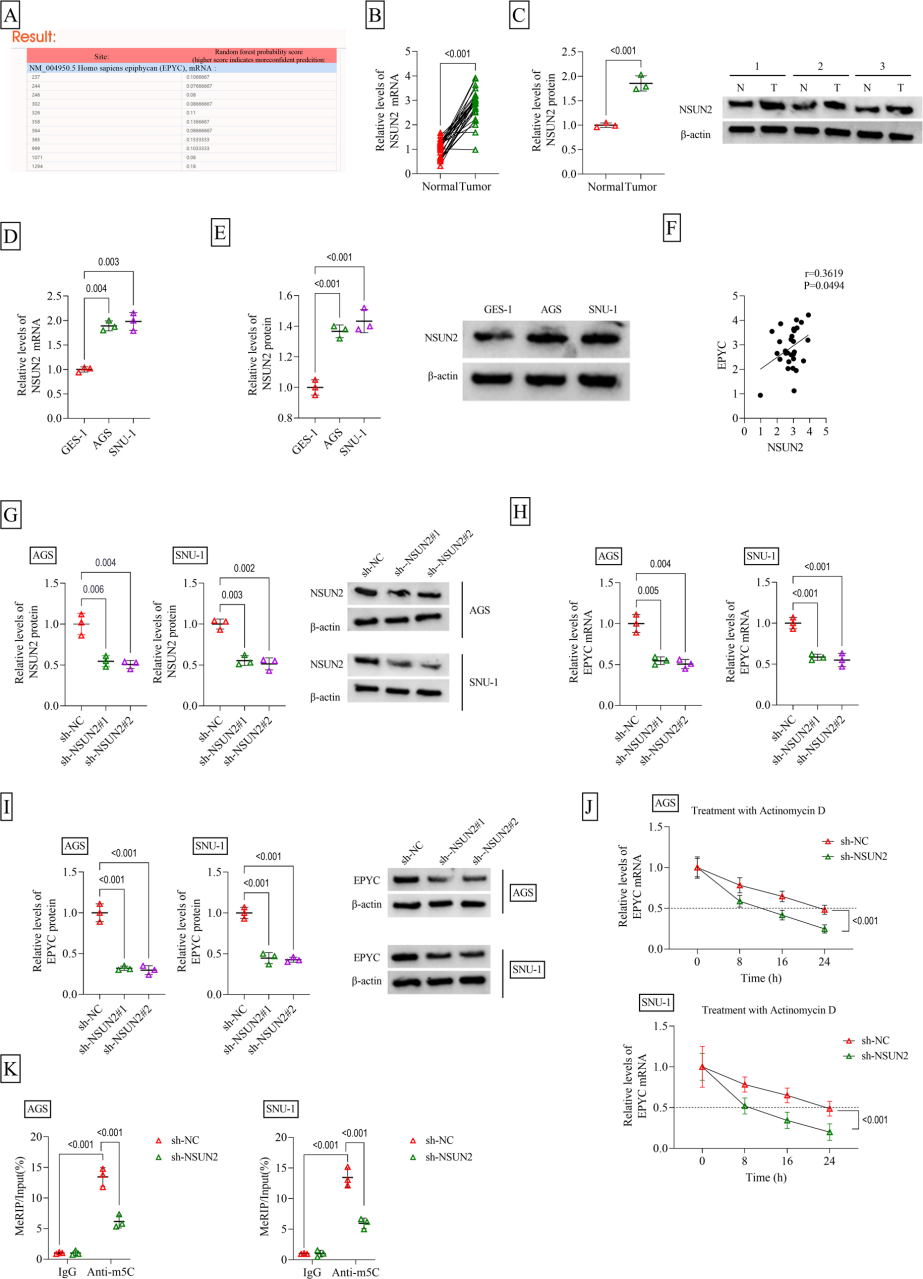
NSUN2 regulated EPYC mRNA stability by m5C modification. **A** RNAm5cfinder software predicted the m5C methylation modification sites of EPYC. **B**-**C** NSUN2 expression in GC tissues and adjacent normal tissues was detected by qRT-PCR (*n* = 30) and WB (*n* = 3). **D**-**E** qRT-PCR and WB were performed to detect NSUN2 expression in GC cells (AGS and SNU-1) and GES-1 cells (*n* = 3). **F** Pearson correlation analysis was used to assess the correlation between NSUN2 and EPYC expression in GC tissues. **G** The transfection efficiency of sh-NSUN2#1/#2 was assessed by WB (*n* = 3). **H**-**I** EPYC mRNA and protein levels were examined by qRT-PCR and WB in AGS and SNU-1 cells transfected with sh-NC/sh-NSUN2#1/#2 (*n* = 3). (J) Actinomycin D treatment assay was used to detect the effect of sh-NSUN2 on the mRNA stability of EPYC (*n* = 3). **K** MeRIP assay was utilized to assess the effect of sh-NSUN2 on the m5C modification of EPYC (*n* = 3). B-C, Student’s *t*-test; D-E and G-I, one-way ANOVA; J-K, two-way ANOVA. All experiments were performed in triplicate

RNAm5cfinder software predicted that EPYC had m5C methylation modification sites (Fig. 4A). NSUN2 was detected to be higher expressed in GC tissues and cells (Fig. 4B-E), and its expression was positively correlated with EPYC expression (Fig. 4F). Besides, TCGA database analyzed NSUN2 expression in different TNM stage and its feasibility as a diagnostic indicator for STAD (Supplementary Fig. 1C-D). To perform further analysis, our study constructed sh-NSUN2#1/#2 and confirmed that they could significantly decrease NSUN2 protein level in AGS and SNU-1 cells after transfection (Fig. 4G). The detection of EPYC mRNA and protein levels suggested that NSUN2 knockdown markedly reduced EPYC expression (Fig. 4H-I). Since the effect of sh-NSUN2#2 was better than that of sh-NSUN2#1, sh-NSUN2#2 (named as sh-NSUN2) was selected for subsequent exploration. Moreover, actinomycin D treatment assay revealed that NSUN2 silencing obviously inhibited the mRNA stability of EPYC (Fig. 4J). Through MeRIP assay, our study determined that downregulation of NSUN2 could significantly decrease the m5C level of EPYC (Fig. 4K). In addition, sh-NSUN2-MUT did not affect the m5C level of EPYC after targeting the 1294 site (higher score) of EPYC (Supplementary Fig. 3 A). Moreover, the m5C level of EPYC was also not affected by sh-NSUN2 after mutation at position 1294 of EPYC (Supplementary Fig. 3B). All data indicated that NSUN2 enhanced the m5C modification of EPYC to promote its expression.

### EPYC overexpression reversed the effect of sh-NSUN2 on GC cell behaviors

**Fig. 5:**
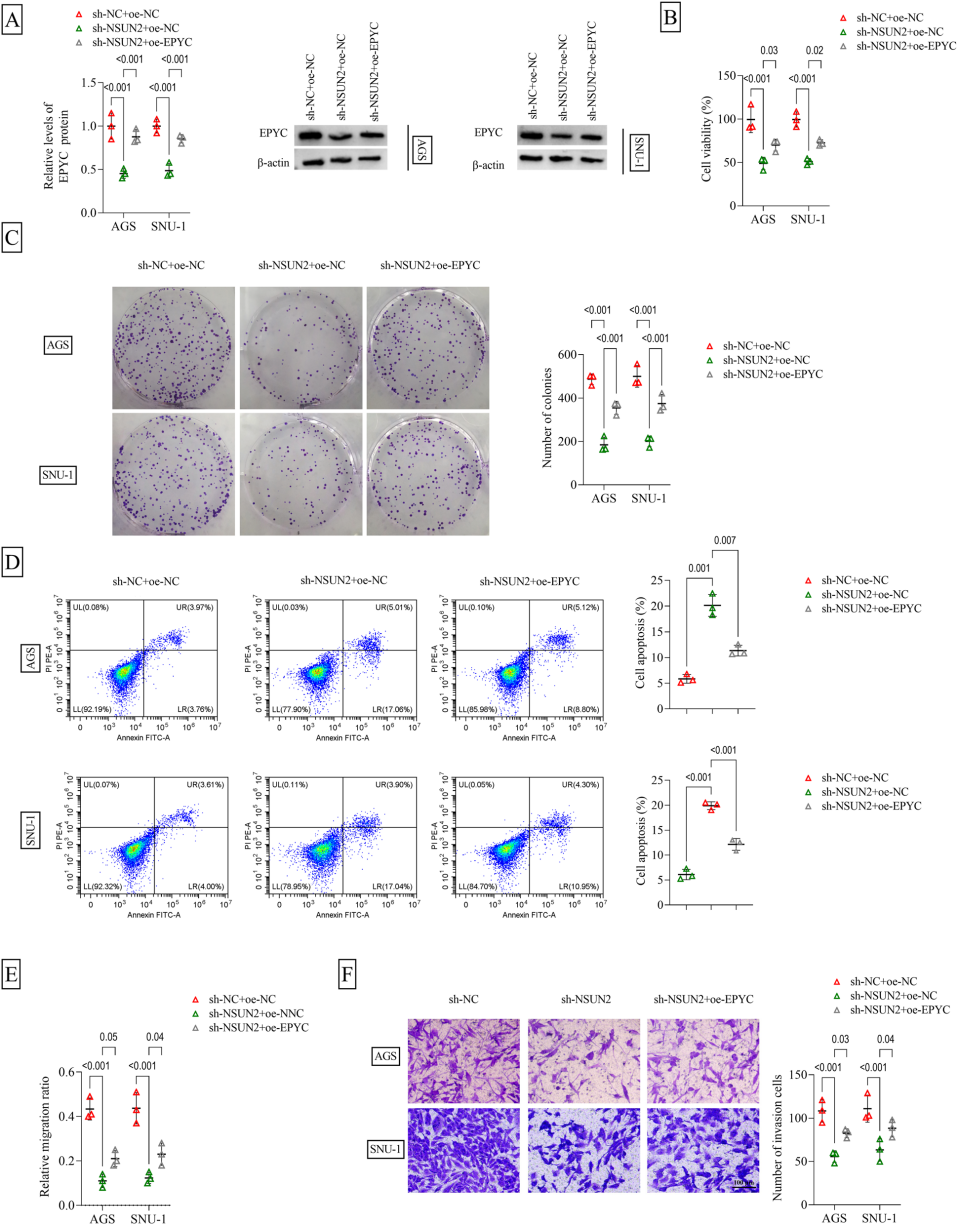
Effect of sh-NSUN2 and oe-EPYC on GC cell proliferation, apoptosis and metastasis. AGS and SNU-1 cells were transfected with sh-NC/sh-NSUN2/oe-NC/oe-EPYC (*n* = 3). **A** WB was used to detect EPYC protein level. Cell proliferation, apoptosis, migration and invasion were determined using **B** CCK8 assay, **C** colony formation assay, **D** flow cytometry, **E** wound healing assay and **F** transwell assay. A-F, two-way ANOVA. All experiments were performed in triplicate

**Fig. 6:**
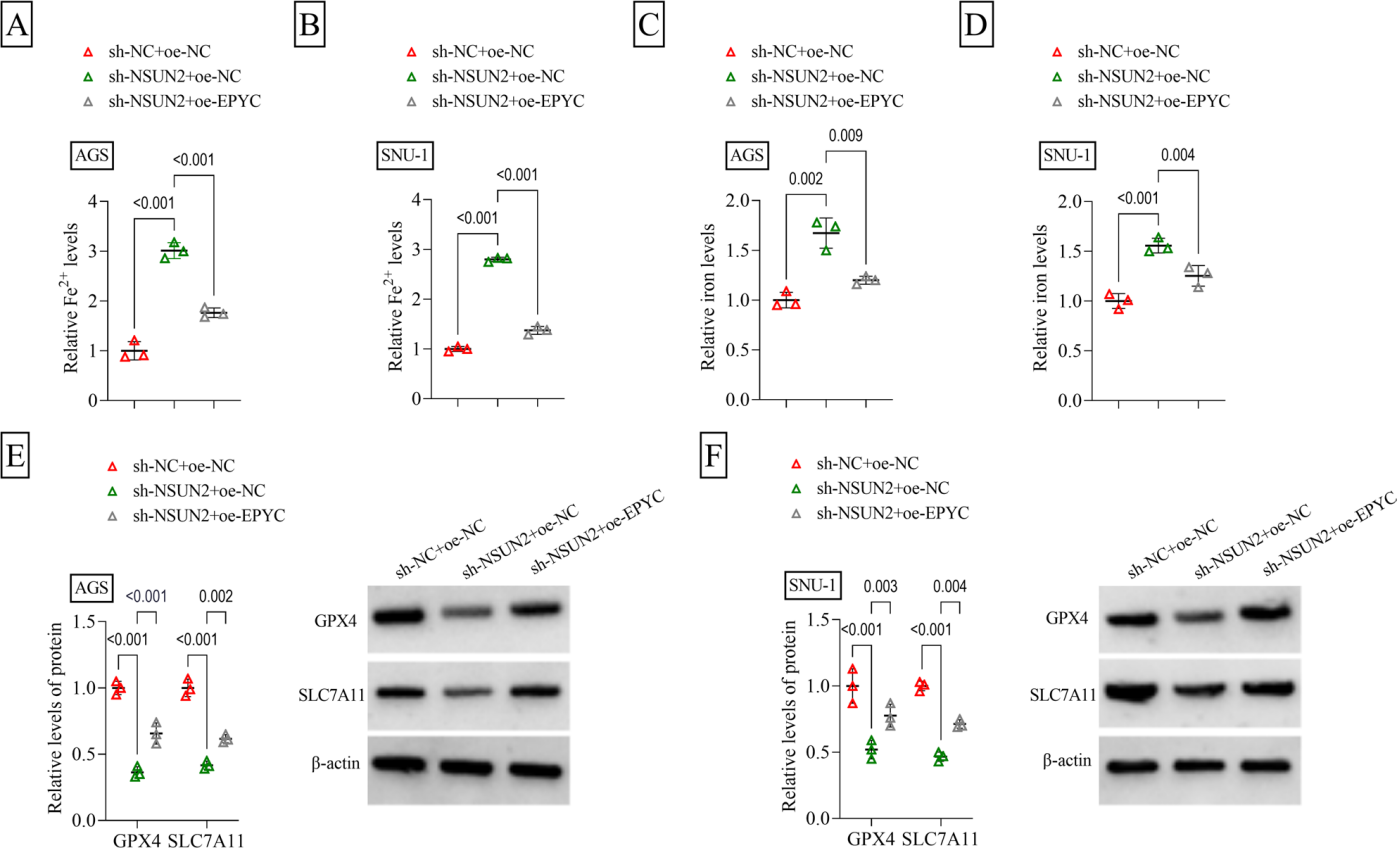
Effect of sh-NSUN2 and oe-EPYC on GC cell ferroptosis. AGS and SNU-1 cells were transfected with sh-NC/sh-NSUN2/oe-NC/oe-EPYC (*n* = 3). **A**-**D** Iron Assay Kit was used to measure Fe^2+^ and iron levels. **E**-**F** WB was used to detect GPX4 and SLC7A11 protein levels. **A**-**F**, two-way ANOVA. All experiments were performed in triplicate

To investigate whether NSUN2 regulated EPYC to mediate GC progression, AGS and SNU-1 cells were co-transfected with sh-NSUN2 and oe-EPYC. The decreasing effect of sh-NSUN2 on EPYC protein level could be recovered by oe-EPYC (Fig. 5A). NSUN2 knockdown suppressed cell viability, decreased colony numbers and enhanced apoptosis rate, while these effects were rescued by EPYC overexpression (Fig. 5B-D). Also, sh-NSUN2-mediated the inhibitory on cell migration ratio and invasion cell number could be abolished by upregulation of EPYC (Fig. 5E-F). In addition, depletion of NSUN2 increased Fe^2+^, iron, lipid ROS and ACSL4 levels, while reduced GPX4 and SLC7A11 protein levels. However, these effects were eliminated by EPYC overexpression (Fig. 6A-F and Supplementary Fig. 2C-D). Moreover, NSUN2 overexpression enhanced colony numbers, reduced apoptosis rate and increased migration ratio in GC cells, while these effects were reversed by EPYC knockdown (Supplementary Fig. 3C-E). To further confirm validate the ferroptosis phenotype, further experiments were performed in this study. CCK8 assay showed that ferroptosis agonist Erastin reduced cell viability in a dose-dependent and time-dependent manner, and the inhibitory effect of Erastin on cell viability was further exacerbated by sh-NSUN2 (Supplementary Fig. 4A-B). Therefore, Erastin treatment with 10 µM for 24 h was chosen for subsequent experiments. Erastin enhanced lipid ROS and ACSL4 levels, while decreased GPX4 level. However, iron chelators DFO and ferroptosis inhibitor Fer-1 had similar effects to oe-EPYC, and both reversed the promotion effect of sh-NSUN2 on lipid ROS and ACSL4 levels, as well as the inhibitory effect on GPX4 level (Supplementary Fig. 5A-B). Above results showed that NSUN2 accelerated GC progression by upregulating EPYC.

### Interference of NSUN2 inhibited GC tumor growth in vivo

**Fig. 7:**
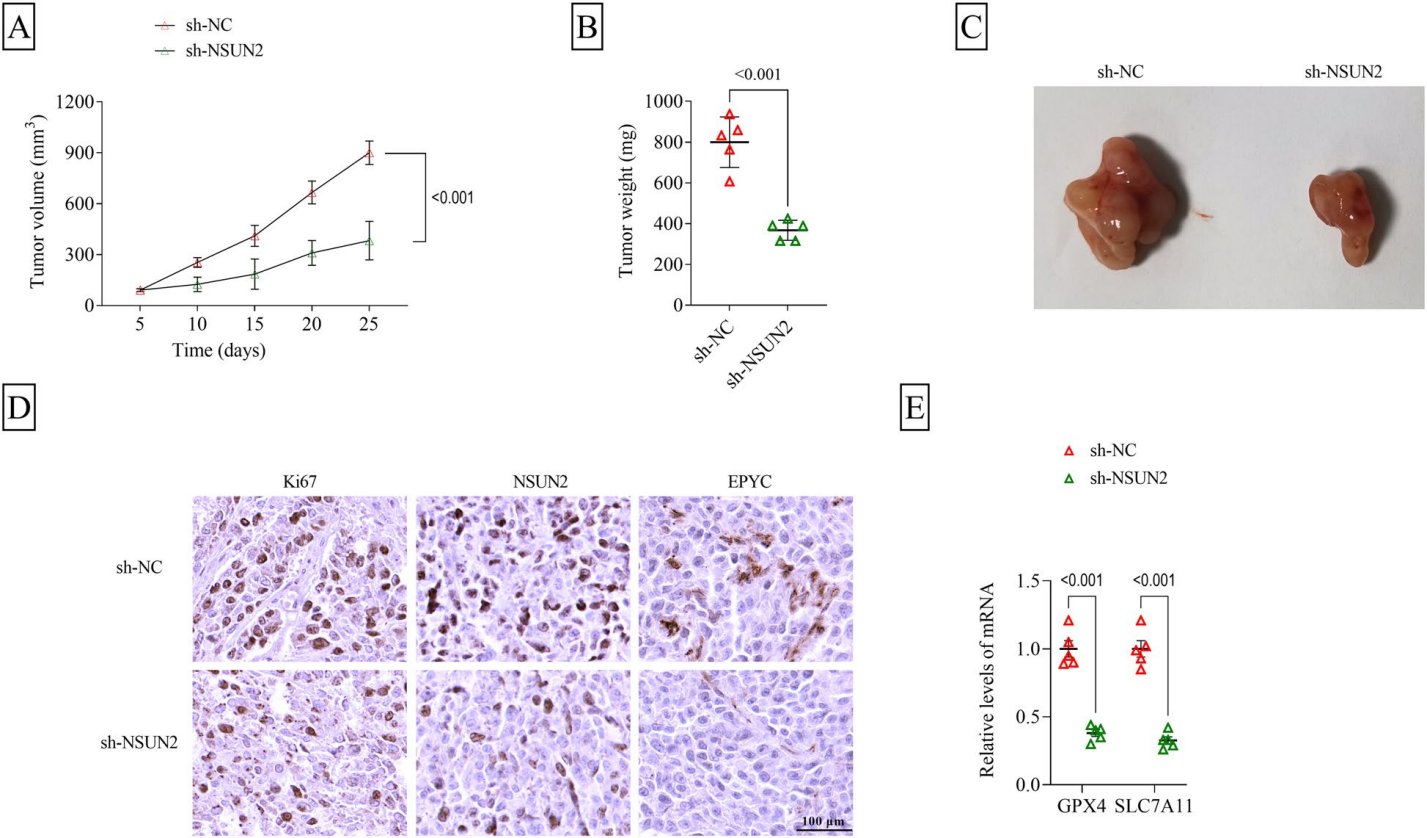
Effect of sh-NSUN2 on GC tumor growth in vivo. AGS cells infected with lentivirus sh-NC/sh-NSUN2 were injected into nude mice (*n* = 5). **A** Tumor volume and **B** weight were detected in each group. **C** Tumor picture in each group is shown. **D** IHC staining was used to examine Ki67, NSUN2 and EPYC positive cells in the tumor tissues of each group. **E** GPX4 and SLC7A11 mRNA levels were detected by qRT-PCR in the tumor tissues of each group. A and E, two-way ANOVA; B, Student’s *t*-test. All experiments were performed in triplicate

Furthermore, animal study was performed to confirm the role of NSUN2/EPYC axis in vivo. Through 25 days of monitoring, our study found that tumor volume, weight and size were markedly reduced in the sh-NSUN2 group (Fig. 7A-C). Besides, our study also detected the decreased Ki67, NSUN2 and EPYC positive cells in the tumor tissues of sh-NSUN2 group using IHC staining (Fig. 7D). The detection of anti-ferroptosis markers suggested that GPX4 and SLC7A11 levels were significantly downregulated in the tumor tissues of sh-NSUN2 group (Fig. 7E). Thus, NSUN2 might increase EPYC expression to accelerate GC tumorigenesis.

## Discussion

It is estimated that the 5-year survival rate for GC is about 32.4%, with the survival rate for advanced GC being even lower [20]. The heterogeneity of GC has unique pathogenesis and active carcinogenic pathway, which makes oncologists often face adverse therapeutic response when treating GC [21]. Recent studies have shown that identifying ideal markers of GC with high sensitivity and specificity will improve the survival rate of patients [22]. Also, targeted therapy is also an effective treatment for GC [21, 23]. Here, our study found that NSUN2-mediated EPYC promoted GC cell malignant behavior, providing a potential molecular target for GC treatment.

EPYC is located on chromosome 12q21.33 and its encoded protein is a part of the extracellular matrix, which is involved in regulating cell behaviors, such as cell adhesion, migration and organizational structure [8, 24]. It had been reported that EPYC was upregulated in osteoarthritis (OA) samples and was associated with immunocyte infiltration levels, which may be a potential biomarker for OA [25, 26]. Besides, EPYC showed higher expression in inner annulus fibrosis cells with degeneration, suggesting that it might be biomarker for intervertebral discs degeneration [27]. Although previous studies have revealed a positive role of EPYC in cancer development [9, 10], its role in GC progression is unknown. Through TCGA, GEPIA and GEO database analysis, our study determined that EPYC was overexpressed in GC tissues, which was also confirmed by further qRT-PCR and WB detection. Functional assay revealed that EPYC silencing could repress GC cell proliferation and metastasis, while accelerate apoptosis and ferroptosis, verifying that EPYC promoted GC cell malignant progression. The above results provide a rationale for EPYC to be a potential target for GC therapy.

NSUN2 belongs to the NSUN RNA methyltransferase family and plays a key role in RNA methylation modification [28]. Previous studies have shown that NSUN2-mediated m5C methylation is involved in regulating various tumors [29]. For example, NSUN2 increased the mRNA stabilization of SKIL by m5C modification, thus enhancing colorectal cancer cell growth [30]. NSUN2 induced m5C modification of FABP5 to increase fatty acid metabolism, cell proliferation and metastasis in osteosarcoma [31]. Importantly, NSUN2 had been confirmed to suppress lipid peroxidation and ferroptosis in endometrial cancer via stimulating SLC7A11 m5C modification [32]. Consistent with previously studies [18, 19], our study also detected the high NSUN2 level in GC tissues. Given the presence of m5C modification sites on the RNA of EPYC, our study evaluated the relationship between NSUN2 and EPYC, and found that NSUN2 could promote the mRNA stability and expression of EPYC by stimulating its m5C modification. Through rescue experiments, our study determined that sh-NSUN2-mediated the inhibiting on GC cell proliferation and metastasis, as well as the enhancing on apoptosis and ferroptosis, were eliminated by EPYC upregulating. Also, NSUN2 interference could reduce the tumor growth of GC via downregulating EPYC level. All data further suggested that NSUN2-activated EPYC facilitated GC cell behaviors and tumorigenesis.

Although our findings shed light on the role of the NSUN2-EPYC axis in GC progression, this study has several limitations. First, the clinical sample size (*n* = 30) was relatively small, and the conclusions need to be verified in larger, multicenter cohorts to increase the generalizability and statistical power. Secondly, our in vivo model mainly demonstrated the role of this axis in tumor growth. However, its effects on metastasis and the tumor microenvironment, particularly the coupling with immune cell infiltration and cytokine networks, warrant further investigation.

Overall, our study revealed that NSUN2 enhanced EPYC mRNA stabilization by m5C modification, thereby facilitating GC cell proliferation, metastasis, repressing apoptosis and ferroptosis. All data provide new insights into the role of NSUN2/EPYC axis, and suggest that NSUN2/EPYC may serve as an underlying therapeutic target for GC patients.

## Supplementary Information

Supplementary Material 1: Supplementary Fig. 1. TCGA database analyzed EPYC/NSUN2 expression and diagnosis value. (A-B) TCGA analyzed EPYC expression in different TNM stage and its diagnosis value for GC patients by ROC curve. (C-D) TCGA analyzed NSUN2 expression in different TNM stage and its diagnosis value for GC patients by ROC curve.

Supplementary Material 2: Supplementary Fig. 2. Effects of sh-EPYC and sh-NSUN2/oe-EPYC on lipid ROS and ACSL4 level. (A) C11-BODIPY probe and (B) WB were used to measure lipid ROS and ACSL4 levels in AGS cells transfected with sh-NC/sh-EPYC (*n *= 3). Lipid ROS and ACSL4 levels in AGS cells transfected with sh-NC/sh-NSUN2/oe-EPYC were determined by (C) C11-BODIPY probe and (D) WB (*n* = 3). A-D, Student’s *t*-test. All experiments were performed in triplicate.

Supplementary Material 3: Supplementary Fig. 3. MeRIP results and the effects of oe-NSUN2/sh-EPYC on GC progression. (A-B) MeRIP assay was used to analyze sh-NSUN2-WT/MUT and sh-NSUN2+oe-EPYC-WT/MUT on the m5C level of EPYC (*n *= 3). (C) Colony formation assay, (D) flow cytometry and (E) wound healing assay were used to detect cell proliferation, apoptosis, and migration in AGS cells transfected with oe-NC/sh-NC/oe-NSUN2/sh-EPYC (*n*=3). A-E, two-way ANOVA. All experiments were performed in triplicate.

Supplementary Material 4: Supplementary Fig. 4. CCK8 screened the optical concentration and time of Erastin in AGS cells. (A) CCK8 assay was used to detect viability in AGS cells treated with different concentrations of Erastin for 24 h and transfected with sh-NSUN2 (*n* = 3). (B) CCK8 assay was used to detect viability in AGS cells treated with 10 µM Erastin for different times and transfected with sh-NSUN2 (*n* = 3). A-B, two-way ANOVA. All experiments were performed in triplicate.

Supplementary Material 5: Supplementary Fig. 5. Effect of Erastin/sh-NSUN2/oe-EPYC/DFO/Fer-1 on lipid ROS, GPX4 and ACSL4 levels. Lipid ROS and GPX4/ACSL4 levels in AGS cells treated with Erastin/DFO/Fer-1 and transfected with sh-NSUN2/oe-EPYC were determined by (A) C11-BODIPY probe and (B) WB (*n* = 3). A-B, two-way ANOVA. All experiments were performed in triplicate.

## Data Availability

The analyzed data sets generated during the present study are available from the corresponding author on reasonable request.
