# Supplementary figures and images for: NSUN2 restrains gastric cancer cell apoptosis and ferroptosis by promoting the m5C modification of EPYC
Source: Hereditas. 2026 Jan 19;163:23. doi: 10.1186/s41065-025-00626-x (PMC12895582; doi:10.1186/s41065-025-00626-x)

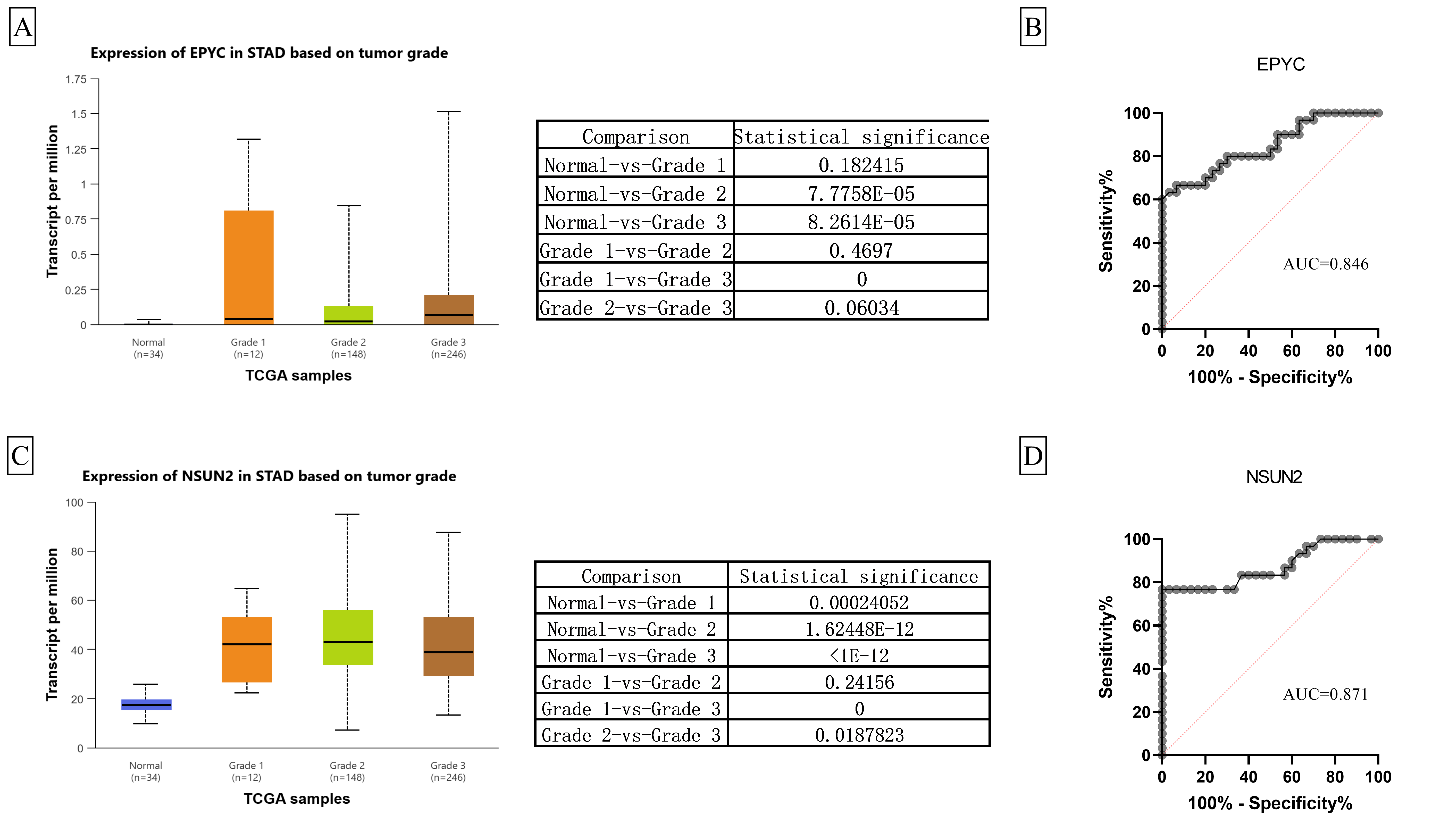

Supplement: Supplementary file 1 — Supplementary Material 1: Supplementary Fig. 1. TCGA database analyzed EPYC/NSUN2 expression and diagnosis value. (A-B) TCGA analyzed EPYC expression in different TNM stage and its diagnosis value for GC patients by ROC curve. (C-D) TCGA analyzed NSUN2 expression in different TNM stage and its diagnosis value for GC patients by ROC curve. [file 41065_2025_626_MOESM1_ESM.tif]

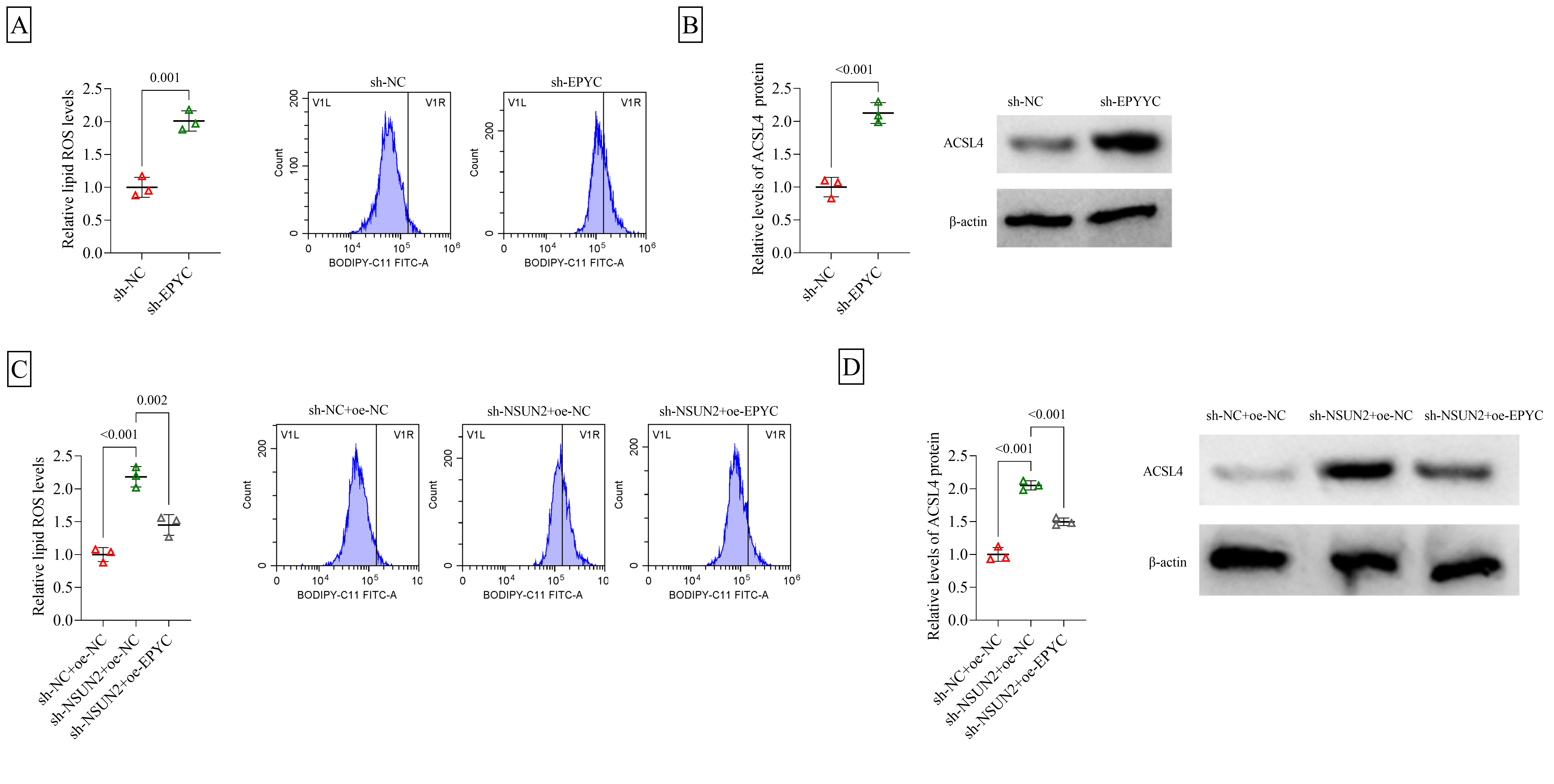

Supplement: Supplementary file 2 — Supplementary Material 2: Supplementary Fig. 2. Effects of sh-EPYC and sh-NSUN2/oe-EPYC on lipid ROS and ACSL4 level. (A) C11-BODIPY probe and (B) WB were used to measure lipid ROS and ACSL4 levels in AGS cells transfected with sh-NC/sh-EPYC (n = 3). Lipid ROS and ACSL4 levels in AGS cells transfected with sh-NC/sh-NSUN2/oe-EPYC were determined by (C) C11-BODIPY probe and (D) WB (n = 3). A-D, Student’s t-test. All experiments were performed in triplicate. [file 41065_2025_626_MOESM2_ESM.tif]

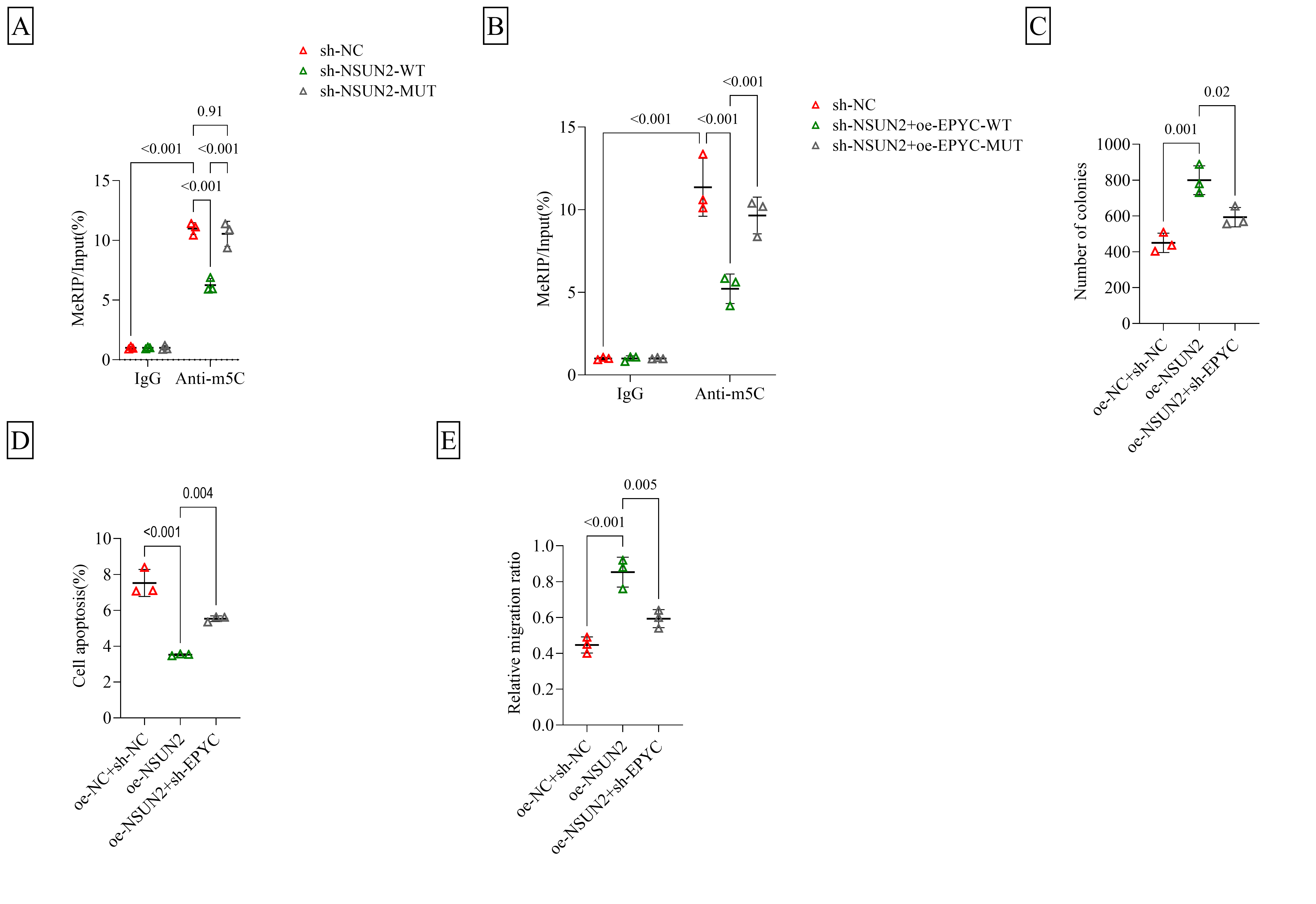

Supplement: Supplementary file 3 — Supplementary Material 3: Supplementary Fig. 3. MeRIP results and the effects of oe-NSUN2/sh-EPYC on GC progression. (A-B) MeRIP assay was used to analyze sh-NSUN2-WT/MUT and sh-NSUN2+oe-EPYC-WT/MUT on the m5C level of EPYC (n = 3). (C) Colony formation assay, (D) flow cytometry and (E) wound healing assay were used to detect cell proliferation, apoptosis, and migration in AGS cells transfected with oe-NC/sh-NC/oe-NSUN2/sh-EPYC (n=3). A-E, two-way ANOVA. All experiments were performed in triplicate. [file 41065_2025_626_MOESM3_ESM.tif]

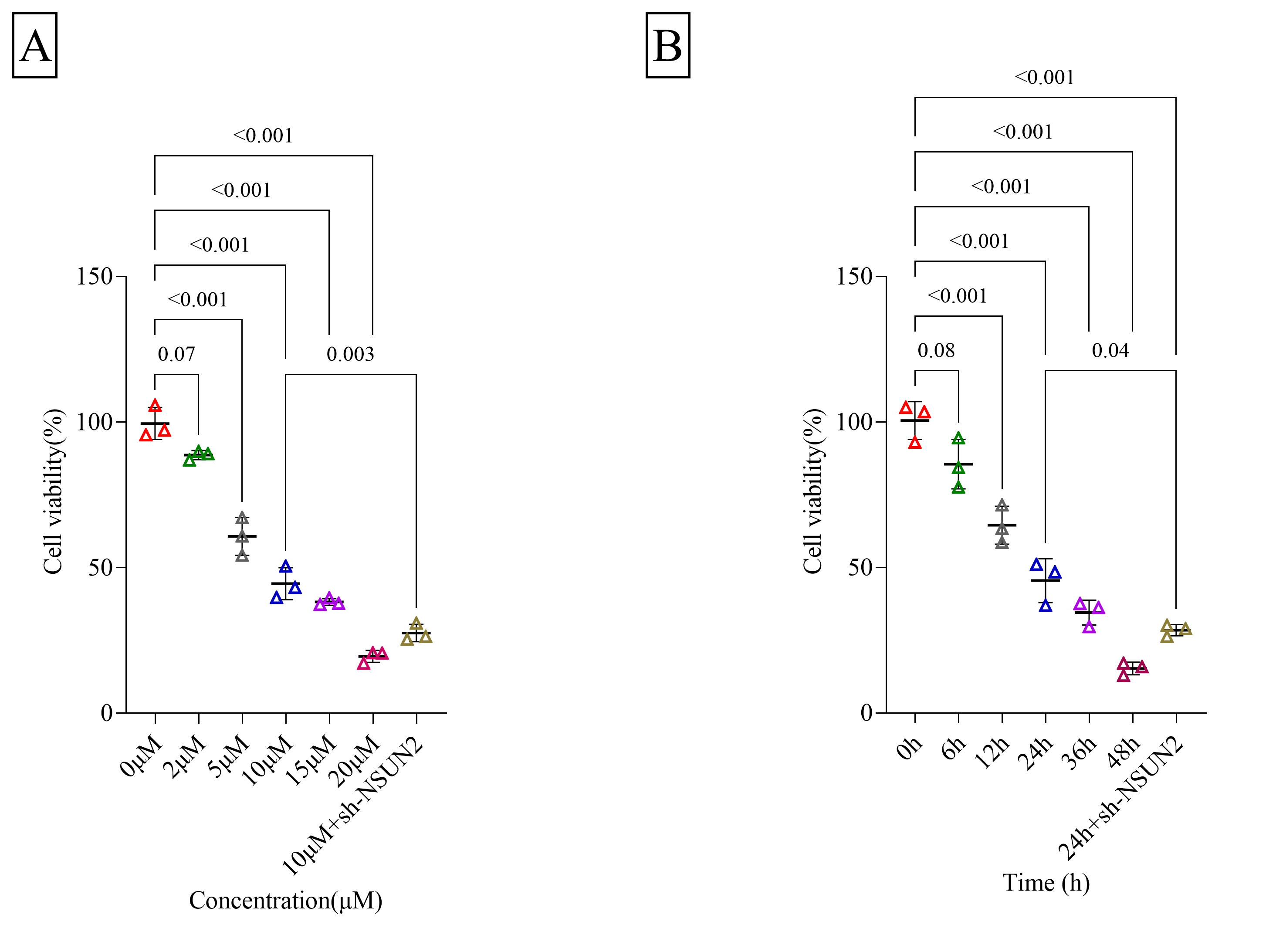

Supplement: Supplementary file 4 — Supplementary Material 4: Supplementary Fig. 4. CCK8 screened the optical concentration and time of Erastin in AGS cells. (A) CCK8 assay was used to detect viability in AGS cells treated with different concentrations of Erastin for 24 h and transfected with sh-NSUN2 (n = 3). (B) CCK8 assay was used to detect viability in AGS cells treated with 10 µM Erastin for different times and transfected with sh-NSUN2 (n = 3). A-B, two-way ANOVA. All experiments were performed in triplicate. [file 41065_2025_626_MOESM4_ESM.tif]

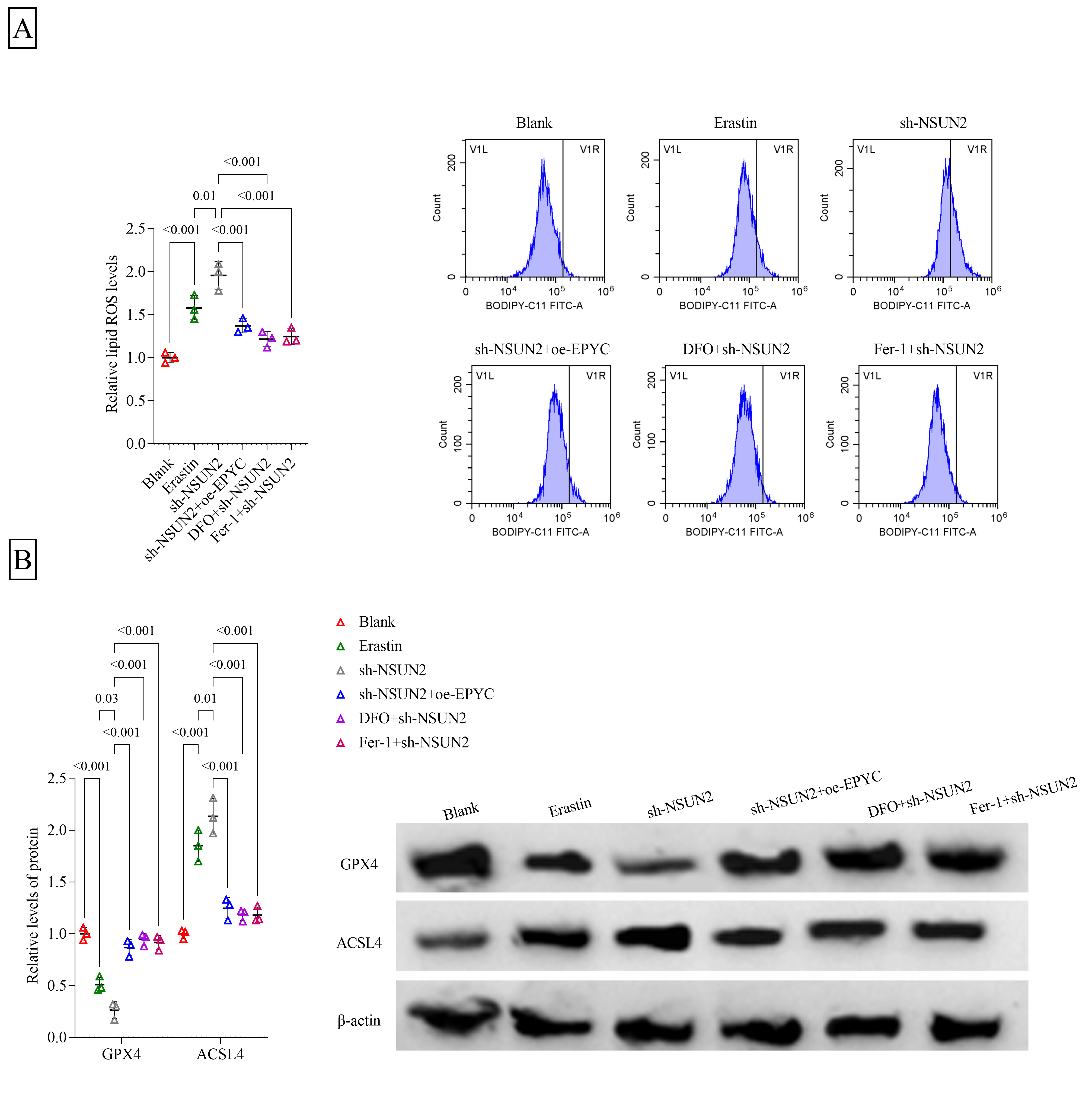

Supplement: Supplementary file 5 — Supplementary Material 5: Supplementary Fig. 5. Effect of Erastin/sh-NSUN2/oe-EPYC/DFO/Fer-1 on lipid ROS, GPX4 and ACSL4 levels. Lipid ROS and GPX4/ACSL4 levels in AGS cells treated with Erastin/DFO/Fer-1 and transfected with sh-NSUN2/oe-EPYC were determined by (A) C11-BODIPY probe and (B) WB (n = 3). A-B, two-way ANOVA. All experiments were performed in triplicate. [file 41065_2025_626_MOESM5_ESM.tif]
